# Critical appraisal of clinical practice guidelines for otitis media in Asian and Western countries with consideration of updates: A narrative review

**DOI:** 10.51866/rv.669

**Published:** 2024-11-29

**Authors:** Nabeel Ibraheem Jaafar Albazah, Hui Min Loh, Jin Yun Lee, Woon Khong Chen, Elena Ying Jie Khaw, Ping Kiat Ngu, Sherreen Yehia Zakaria Elhariri

**Affiliations:** 1 MBBCh, MSc, FRCS, Department of Surgery, IMU University, clinical campus Seremban, Jalan Rasah, Seremban, Negeri Sembilan, Malaysia. Email: sherreenelhariri@imu.edu.my; 2 MBCHB, M.Med ORL-HNS, Department of Surgery, International Medical University, Clinical campus, Seremban, Malaysia.; 3 MBBS, Sultanah Nora Ismail Hospital (HSNI), Batu Pahat. Malaysia.; 4 MBBS, Sultanah Nora Ismail Hospital (HSNI), Batu Pahat. Malaysia.; 5 MBBS, The Tengku Ampuan Rahimah Hospital in Klang. Malaysia.; 6 MBBS, Hospital Sultan Abdul Halim. sungai petani Kedah, Malaysia.; 7 MBBS, Hospital Sultan Idris Shah, Serdang, Malaysia.

**Keywords:** Otitis media with effusion, Guidelines, Management, Children

## Abstract

**Introduction:**

Acute otitis media is a common disease in children under the age of 5 years due to their primitive ear anatomy. One of its consequences is otitis media with effusion (OME), which is a condition wherein serous secretion accumulates in the middle ear due to Eustachian tube dysfunction. OME results in conductive hearing loss, impacting children’s learning and development. This review aimed to identify discrepancies in clinical practice guidelines (CPGs) for managing OME in children across Asian and Western countries, focusing on updates to these guidelines.

**Methods:**

A comparative review involving critical appraisal using the Appraisal of Guidelines for Research and Evaluation II tool was conducted. CPGs for managing ear effusion in children in Western and Asian countries published in the PubMed, Cochrane Library, Scopus and Science Direct databases were reviewed. Four CPGs from Western countries (Scotland, England, the United States of America and France) and three CPGs from Asian countries (Japan, Korea and Malaysia) were selected.

**Results:**

There was a mild discrepancy regarding conducting surgery after 3 months, particularly myringotomy and adenoidectomy with or without grommets, between the Scottish CPG and other CPGs.

**Conclusion:**

Asian CPGs for the management of OME share many similarities with Western CPGs. However, Asian CPGs tend to be stronger in most domains and levels of evidence. Japanese and American CPGs are up to date as of 2022 and 2016, respectively, while other CPGs are outdated for about 10 years.

## Introduction

According to the Institute of Medicine, clinical practice guidelines (CPGs) include recommendations aimed at enhancing patient care based on a systematic review of evidence and an assessment of the advantages and disadvantages of alternative care options.^1^ These guidelines are developed by a multidisciplinary team, aiming to promote excellence in managing conditions and providing a road map for physicians and medical practitioners to follow. Additionally, CPGs seek to improve the accuracy of diagnosis and treatment.^2^

### Definition of otitis media with effusion (OME)

Otitis media is an infection of the middle ear, located between the tympanic membrane laterally and the inner ear medially, where the ossicular bony chain is situated. Acute otitis media (AOM) represents a significant proportion of childhood illnesses, especially among children less than 5 years old, and is the most common reason for visiting primary care clinics and receiving antibiotics.^3,4^

In developed countries, the incidence of AOM reaches up to 80% in children by the age of 3 years. One of the common complications of middle ear infection is OME, wherein fluid accumulates in the middle ear because of inflammation. This fluid accumulation disrupts the normal conduction of sound waves in the middle ear and results in conductive hearing loss. This condition is especially common in young children due to their primitive Eustachian tube anatomy as well as their general increased susceptibility to upper respiratory tract infections, which are a known precedent to AOM. Abnormal Eustachian tube function and upper airway anatomy are also responsible for susceptibility to OME in children with genetic anomalies such as Down’s syndrome and congenital anomalies such as a cleft palate.^3,4^

### Prevalence of OME

OME accounts for up to 90% of paediatric illnesses, affecting children at least once during their preschool years. It is also the most common cause of hearing impairment in this age group. During the first year of life, OME accounts for 50% of paediatric illnesses, increasing to 60% by the age of 2 years. Most cases recover completely within 3 months without active intervention. However, 5%–10% of children may experience a longer duration of OME, lasting up to 1 year or more, which requires special attention and prolonged medical treatment. Additionally, 30%-40% of cases may recur.^5^

OME is a significant childhood illness, as conductive hearing loss can negatively impact children’s development, especially in the first 6-12 months of life.^6^ Moreover, the combination of AOM and OME substantially affects the quality of life of both children and their parents, incurring physical, emotional and financial costs.^7^

In this study, CPGs for managing OME in four Western countries and three Asian countries were critically appraised using the Appraisal of Guidelines for Research and Evaluation (AGREE) II tool to identify their strengths and reliabilities. A comparative review was also conducted. We believe there may be discrepancies in the CPGs for managing OME because different continents have different racial and environmental factors. We found that the American CPG has been reviewed and updated multiple times and therefore considered this guideline as a standard.^2,8-10^

### AGREE II tool

The AGREE II tool is widely used for assessing the quality of CPGs. Regarding content validity, the tool has been developed through a rigorous process involving extensive literature reviews and expert consultations to ensure comprehensive coverage of guideline quality.^11^ Regarding construct validity, it effectively differentiates between high- and low-quality guidelines.^12^ The AGREE II tool also demonstrates high inter-rater reliability, indicating that different evaluators consistently produce similar assessments.^13^ However, some users may find certain items subjective, potentially leading to variability in scoring despite the tool’s high overall reliability. To assess the AGREE II score, the principal investigator registered the application and invited two other reviewers for this study. The tool comprises six domains with varying maximum scores: Domains 1, 2 and 4 have a maximum score of 42; Domain 3, 112; Domain 5, 56; and Domain 6, 28. Conversely, the minimum scores are 6 for Domains 1, 2 and 4; 16 for Domain 3; 8 for Domain 5; and 4 for Domain 6, with no established cut-off score. The score ranges from 1 (strongly disagree) to 7 (strongly agree) based on the appraisal choices. For each item, the score is calculated by subtracting the minimum score from the maximum score. For example, with a maximum score of 42 and a minimum score of 6 for Domain 1, the obtained score is 39. The percentage for Domain 1 is calculated using the following formula: [(obtained score - minimum score)/(maximum score - minimum score)] × 100. Thus, for Domain 1, the percentage is calculated as [(39-6)/42-6)] × 100=91%.

## Methods

### Objectives

The general objective of this review was to identify discrepancies in CPGs for managing OME in children across Asian and Western countries, with a focus on updates to these guidelines. The specific objectives were to critically appraise existing and well-established CPGs for the management of OME in children, to compare the recommendations provided in CPGs from Asian and Western countries regarding the management of OME in children, to identify any disparities between such recommendations and to provide recommendations for harmonising or adapting CPGs to better suit the needs of diverse populations or healthcare systems and determine whether the Malaysian CPG needs to be updated.

### Data sources and search strategy

We conducted a qualitative study of selected Asian CPGs available online describing the diagnosis and management of OME in children and compared them to Western CPGs. We searched online databases such as PubMed, Cochrane Library, Ovid, Scopus and Science Direct using the following keywords: ‘otitis media with effusion’, ‘guidelines’, ‘management’ and ‘children’.

### Inclusion criteria

All CPGs published online from 2012 onwards regarding the management of OME in Western countries including England, Scotland, France and the United States of America (USA) and Asian countries including Japan, Malaysia and Korea were included in the study.

### Exclusion criteria

Case reports, systematic reviews, randomised controlled trials and meta-analyses about the topic; similar reviews not representing the CPG of a specific country; and CPGs in languages other than English were excluded from the study.

### Study selection and quality assessment

We reviewed all relevant database search results and scanned the text, including references, for any material referencing CPGs or providing direct links to CPGs. From these materials, we extracted links to the online versions of the CPGs from various countries for further analysis. We selected only the latest versions of the CPGs and excluded those that were not published in English. Updates to each country’s CPG were also reviewed.

### CPG review process

Two investigators independently extracted data from the included studies, and disagreements between them were resolved via consensus.

### Data extraction and synthesis

Among the selected well-established CPGs in both Asian and Western countries published online, details on the diagnosis (screening, diagnostic criteria and diagnostic tests) and management (watchful waiting, conservative and surgical) of OME among children and those with special needs were extracted. Based on the results, a thematic analysis was conducted, and the CPGs were categorised according to their management approach by tabulating and comparing the data based on the diagnosis and management of OME in children and those with special needs.

### Quality assessment

The AGREE II tool was used to evaluate the quality of the CPGs, focusing on their accuracy and clarity throughout the development process (http://www.agreetrust.org). It consists of six domains and 23 items (Table 1). The AGREE II score ranges from 1, indicating no information in the guideline, to 7, indicating exceptional reporting. In this study, all scores were summed by two reviewers, and the percentage was calculated.

**Table 1:** AGREE II^*^ scores (%) of the Western and Asian CPGs for OME management.

No.	Domain name	Western countries (Scotland, England, France and the USA)^**^	Asian countries (Japan, Korea and Malaysia)^***^	Range
1	Scope and purpose	82.5	90.0	10-100
2	Stakeholder involvement	74	88.3	0-92
3	Rigour of development	72.7	82.0	0-83
4	Clarity of presentation	93.7	99.0	21-100
5	Applicability	71	84.3	0-85
6	Editorial independence	63.5	90.3	0-96
	Total for all domains	77.1	88.7	

* AGREE: Appraisal of Guidelines for Research and Evaluation

** Year of the study: Scotland (2003), England (2008), France (2018) and the USA (2013)

*** Year of the study: Japan (2015), Korea (2012) and Malaysia (2012)

The maximum score was calculated as follows: 7 (strongly agree) ×3 (items) ×2 (number of appraisals in our study) = 42. Conversely, the minimum score was calculated as follows: 1 (strongly disagree) ×3 (items) ×2 (number of appraisals in our study) = 6.

The results of the quality assessment are shown in Table 1. The total AGREE II score of the CPGs from the Western countries was 77.1%. Specifically, the scores of the CPGs from Scotland, England, France and the USA were 62.8%, 88%, 78.6% and 79.15%, respectively. Conversely, the total AGREE II score of the CPGs from the Asian countries was 88.7%. The scores of the CPGs from Korea, Malaysia and Japan were 85.4%, 90.13% and 90.65%, respectively. The overall score of all CPGs from both Western and Asian countries was 82.9%. The AGREE II score of the Asian CPGs was approximately 12% higher than that of the Western CPGs.

The level of evidence in the CPGs from the Western and Asian countries is shown in Table 2. The French CPG demonstrated the lowest level of evidence.

**Table 2:** Level of evidence in the CPGs from the Western and Asian countries.

Level of evidence	Scotland	England	France	Japan	Korea	Malaysia	USA
Consideration of benefits and harms (AGREE II **Item 11**)	7	6	4	7	6	6	7
Strengths and limitations of the evidence (AGREE II **Item 9**)	6	5	1	3	7	6	5
Link between recommendations and evidence (AGREE II **Item 12**)	6	6	4	6	6	6	7

## Results

### Study characteristics

The characteristics of the seven included studies are shown in Tables 3–5. The studies were conducted in Japan (2015), Malaysia (2012), Scotland (2003), England (2008), France (2018), Korea (2012) and the USA (2013).^5,6,11-16^

**Table 3:** Diagnostic and management criteria for OME in Scotland, England and France.

Diagnostic criteria		
Scotland^14^	England^15^	France^16^
Audiometry Tympanometry Tuning fork test Otoscopy - At least two tympanic membrane abnormalities: abnormal colour such as yellow, amber or blue; opacification other than that due to scarring; and decreased or absent mobility - Retracted/concave tympanic membrane with a colour change (typically yellow or amber), air bubbles, air-fluid level, fullness or bulging - Reduced or absent mobility on pneumo-otoscopy	**Clinical examination Otoscopy** Otoscope, general upper respiratory health, development of the condition **Hearing test Tympanometry**	**Otoscopy and tympanometry** - Otoscopy alone is suggestive. - Pneumatic otoscopy **Nasal endoscopy** - Unilateral OME - Mouth breathing or snoring when adenoidectomy without tympanostomy tube placement is predicted **Pure-tone audiometry** - Speech and hearing assessment - Auditory evoked potentials or auditory steady-state responses **Assessment of gastro-oesophageal reflux disorder** - Considered in severe forms of OME in children aged >7 years - Relevant in cases of OME combined with other diseases, such as recurrent laryngitis or recurrent rhinosinusitis **Speech therapy assessment** - Conducted for children with language delays, speech delays or reading difficulties

Management criteria: Conservative			
	Scotland	England	France
**Antibiotics**	Not recommended	Not recommended	- Slight impact on retro-tympanic effusion - Does not increase medium- or long-term hearing - Possibility of adverse effects and bacterial resistance
**Antihistamines and decongestants**	Not recommended	Not recommended	- Look to be effective in the medium- or long-term resolution of retro-tympanic effusion
**Nasal or oral steroids**	Not recommended	Not recommended	- May be momentarily effective - Does not enhance the resolution of OME or medium- or long-term hearing
**Others**	**Mucolytics** - Not recommended **Auto inflation** - May be of benefit to some children **Homeopathy** - No data available to make any recommendations	**Hearing aids** - Recommended to children with persistent bilateral OME and hearing loss as a substitute for surgical intervention where surgery is contraindicated or not appropriate **Auto inflation** - May be considered during the active observation period for children who are expected to cooperate	**Mucolytics** - Can enhance OME in one-third of children, but with no proven long-term efficacy **Pressure treatment** - Valsalva manoeuvre or balloon tubal insufflation **Thermal treatment** - Standard aerosol therapy **Sulphurous water**
**Watchful waiting**	**If the patient is not in danger** - Patient and family education is provided, and watchful waiting is done. - Reassessment 3 months later - Reevaluate until effusion is no longer present, considerable hearing loss is identified, or structural malformation of the eardrum or middle ear is suspected.	The persistence of bilateral OME and hearing loss should be validated for 3 months before starting the intervention. Child hearing should be retested at the end of this period.	**Not mentioned**

**Table 4:** Diagnostic and management criteria for OME in Japan, Korea and Malaysia.

Diagnostic criteria		
Japan^5^	Korea^17^	Malaysia^1,6^
**Otoscopy** - Retraction, bulging or opacity of the membrane - Diminished or absent light reflex - Presence of middle ear effusion - With or without variously coloured middle ear effusion **Pneumatic otoscopy** - Diminished or reduced mobility of the tympanic membrane - Observe in detail using an operating microscope, an otoscope or a pneumatic otoscope **Pure-tone audiometry** - For diagnosing the severity and type of hearing loss - Performed when confirming hearing loss before and after tympanostomy tube insertion **Tympanometry** - Assesses changes in the compliance of the tympanic membrane and middle ear **Imaging of the temporal bone** - Helpful for estimating the development of mastoid cells	Otoscopy, pneumatic otoscopy, otoendoscopy, operating microscopy and tympanometry type B/C are used to evaluate the existence of middle ear fluid. **Otoscopy** - Air–fluid level, air bubbles, retraction, atelectasis, hyperaemia and colour change (e.g. amber, brownish or dark blue) **Pneumatic otoscopy Otoendoscopy** **Operating microscopy Tympanometry** - OME can be proposed based on type B or C tympanogram **Audiometry** - Behavioural observation audiometry for children under 6 months old - Visual reinforcement audiometry for children 6–24 months old - Play audiometry for children 24–48 months old - Standard pure-tone audiometry in children >4 years old - Auditory brainstem response or distortion-product otoacoustic emission audiogram may be utilised to estimate hearing threshold.	The following methods are used for the diagnosis of OME: pneumatic otoscopy tympanometry acoustic stapedial reflex testing otoacoustic emission audiometry

Management criteria: Conservative			
	Japan	Korea	Malaysia
**Antibiotics**	**Antibiotics** - In cases without infection of the adjacent organs, administration of antibacterial agents for OME in children is inadvisable. Macrolide treatment is therapeutic. - Preference in children with OME associated with rhinosinusitis	Not recommended	Not recommended
**Antihistamines and decongestants**	Should be counted as an option for patients with allergic rhinitis	Not recommended	Not recommended
**Nasal or oral steroids**	Not recommended	Not recommended	**Oral steroids** - Not recommended **Intranasal steroids** - Used less than 6 weeks for OME in cases of concurrent allergic rhinitis and adenoid hypertrophy
**Others**	**Mucolytics** - Carbocysteine is the only oral medication currently authorised in Japan for OME in children and is supposed to be effective for inflammatory lesions of the surrounding organs.	**Auto inflation** - May be recommended in cooperative children **Hearing aids** - Considered for children whose surgical approach may not be feasible for reasons such as other medical problems or lack of consent by parent or caregiver	**Auto inflation homeopathy and mucolytic** - Not recommended
**Watchful waiting**	**OME lasting >3 months** - Watchful waiting for 3 months from the time of effusion onset or the day of diagnosis of OME without any pathological changes in the eardrum - Continue close observation beyond 3 months.	**For patients with non-high-risk factors** - Watchful waiting for 3 months unless cases necessitate earlier intervention when a permanent change in the tympanic membrane is expected	**Active observation** - Adopted for 3 months before surgery, along with education to minimise the impact of hearing loss during this period

**Table 5:** Diagnostic and management criteria for OME in the USA.

Diagnostic criteria	Management criteria: Conservative	Management criteria: Surgical
**Pneumatic otoscopy** - Backbone for the diagnosis of OME in children with otalgia, hearing loss or both - The movement of the tympanic membrane is sluggish, stifled or restricted; a complete absence of mobility is not required. **Tympanometry** - Confirmation of OME (when the diagnosis is doubtful after performing or attempting pneumatic otoscopy) - Atypical tympanometric width (>250 daPa) combined with low peak admittance - Width measurement (type B or broad tympanogram) to peak admittance (type AS or shallow tympanogram) - In infants < 6 months of age, a higher-frequency probe tone (1000 Hz) is indicated. - Conduct an age-appropriate hearing test if OME persists for 3 months or any duration in an at-risk child.	**Antibiotics, antihistamines, decongestants and nasal or oral steroids** - Not recommended **Other high-risk patients should** undergo a hearing test. If the tympanogram shows type B findings, proceed to watchful waiting. **Watchful waiting** - If the patient is not in danger, patient and family education is provided, and watchful waiting is done. - Reassessment 3 months later - Reevaluate until effusion is no longer present, considerable hearing loss is identified, or structural malformation of the eardrum or middle ear is suspected.	Type B tympanogram in at-risk patients who have OME in either ear or offer surgery. **Children aged <4 years** - Advise for tympanostomy tube placement. - Adenoidectomy is indicated in case of conditions other than OME (e.g. nasal obstruction or chronic adenoiditis) **Children aged >4 years** - Advocate tympanostomy tube placement with or without adenoidectomy. **Children with special needs including those with Downs syndrome** - Hearing assessments are suggested every 6 months starting at birth. - An otolaryngologist evaluation is recommended if the middle ear status is uncertain or if hearing loss is found. **Children with a stenotic ear canal** - Should be reviewed with otologic microscopy every 3-6 months to remove cerumen and detect OME **Children with a cleft palate** - Screening for OME and hearing loss should continue throughout childhood, including after palatal repair.

### Management criteria: Surgical treatment in the Western countries

In Scotland, children with persistent OME lasting more than 3 months and those with speech and language, developmental or behavioural challenges should be referred to an otolaryngologist. Insertion of a ventilation tube may assist with expressive language and verbal comprehension; however, the timing of surgery is not critical. The management of children with special needs is not mentioned.

In England, children with persistent bilateral OME documented for 3 months and a hearing level in the better ear of 20-30 dB HL or averaging 0.5, 1, 2 and 4 kHz should be considered for surgical management. Surgical intervention may also be considered in children with obstinate bilateral OME and a hearing level below 25-30 dB HL, particularly if the hearing loss significantly affects their developmental, social or learning status. Ventilation tube placement and adenoidectomy are not recommended in the absence of constant and/or frequent upper respiratory tract infections.

Children with Down’s syndrome or a cleft palate should be monitored regularly for OME. A hearing aid is recommended for children with Down’s syndrome and OME with hearing loss, but a ventilation tube can be offered as a substitute after consideration of the severity of hearing loss, age, usefulness, risks and the possibility of early extrusion of the ventilation tube. Conversely, tube insertion and primary cleft palatal repair must be performed in children with a cleft palate with OME and constant hearing loss.

In France, tympanostomy tube placement or adenoidectomy with tympanostomy tube placement is implemented only in children under the age of 4 years when there is symptomatic adenoid hypertrophy (e.g. nasal obstruction and recurrent infections). Myringotomy with adenoidectomy is useful in the management of OME but appears to be inferior to tympanostomy tube placement in terms of 6-month quality of life. The use of a mastoid antrum ventilator tube lacks evidence and is therefore not recommended. There is no specific mention of guidelines for children with special needs.

The CPG applied in the USA is shown in Table 5.

### Management criteria: Surgical treatment in the Asian countries

In Japan, tympanostomy is suggested for the management of bilateral OME lasting more than 3 months, even in cases with hearing loss of 2539 db. Short-term tubes (8-16 months) should be the first option for initial tube insertion in children without a risk of becoming refractory. Long-term tubes (18-36 months) should be considered for repeat surgery in cases of recurrent refractory OME. They may also be indicated in the presence of pathological changes such as atelectatic ear and adhesive otitis media, which are prone to premature extrusion of short-term tubes.

Adenoidectomy is effective for the treatment of OME but is not recommended as the primary procedure for OME in children without clear indications related to upper airway lesions. Once the absence of a cleft palate is confirmed, adenoidectomy can be performed as a repeat surgery in cases of recurrent OME after initial tympanostomy tube insertion.

Children with special needs such as those with Down’s syndrome and a cleft palate are categorised as a high-risk group. Thus, all recommendations for high-risk groups apply to them.

In Korea, early intervention is crucial. Urgent surgical intervention is advised for children at a high risk or those showing changes in the tympanic membrane during the 3-month observation period that may lead to irreversible changes, such as retraction pockets, cholesteatoma, ossicular erosion and adhesive otitis media. For otherwise healthy children, surgical intervention is recommended for bilateral hearing loss if the hearing level in the better ear is 40 dB HL or above. If the hearing level ranges from 20 to 40 dB HL, the guideline suggests considering children’s medical and developmental status and parents’ preferences. For unilateral hearing loss, physicians may consider surgery based on the disease duration, hearing level and parental preference. Ventilation tube placement is the desired method for initial surgical intervention. For recurrent cases, ventilation tube placement with adenoidectomy and/or tonsillectomy may be indicated for repeated surgical intervention.

For children with Down’s syndrome, craniofacial anomalies or a cleft palate, a thorough assessment of the medical and developmental status is essential. Age-appropriate hearing testing and active intervention for associated hearing loss should be conducted. Surgical intervention may be performed earlier, and hearing aids may be provided based on the hearing status, regardless of whether surgical intervention is undertaken.

In Malaysia, myringotomy with insertion of ventilation tubes is indicated for conductive hearing loss greater than 25 dB persisting for more than 3 months. Adenoidectomy is considered in the presence of hypertrophied adenoids abutting the torus tubarius

For children with cleft lip and palate, myringotomy and ventilation tube insertion are performed at the time of palatal repair. For those with Down’s syndrome, the procedures are conducted after weighing the risks and benefits, provided that only OME exists. Hearing amplification may be considered in cases of mixed or moderate hearing loss in both children with a cleft palate and Down’s syndrome.

## Discussion

Among the three Asian countries including Malaysia, Korea and Japan and four Western countries including Scotland, France, England and the USA, only the USA has an established screening procedure for OME. The USA implements a universal hearing screening programme, which is also available in Malaysia. This programme is designed to detect hearing loss in newborn babies before 3 months of age and to provide intervention no later than 6 months of age.^18^ Furthermore, the programme aims to detect congenital or early hearing loss in children,^19^ which contrasts with issues related to OME.

OME is a common cause of acquired conductive hearing loss in childhood, typically affecting children between the ages of 1 and 6 years, with a higher prevalence at 2 years of age.^20^ This finding raises concerns about the effectiveness of the universal hearing screening programme in detecting OME. Currently, the only established screening tool for OME in the above-mentioned countries relies on parental suspicion of hearing impairment. However, the sensitivity of this approach is low, with only 19.7% of cases being detected, leaving 80% of cases missed.^21^ This is concerning, as different countries have varying criteria for diagnosing OME.

### Diagnostic criteria

**Asian countries**

Malaysia’s diagnostic criteria for OME include relevant history suggestive of the condition, such as hearing impairment, speech or language difficulties and poor school performance. These criteria can be supplemented with clinical examinations and additional tests, such as the tuning fork test, otoscopy or pneumo-otoscopy and tympanometry screening.

In contrast to Malaysia, Japan has established criteria for the diagnosis of OME, categorised into acute, subacute and chronic phases, corresponding to durations of 3 weeks, 1-3 months and more than 3 months after the onset of the disease, respectively. Japan also classifies the fluid into serous, viscous and mucopurulent and considers patient history in differentiating OME from AOM. Conversely, the only criterion for diagnosis in Korea is the establishment of fluid presence through examination.

**Western countries**

Scotland and England are similar to Malaysia concerning the relevant history and examination for diagnosing OME. France and the USA include an additional diagnostic criterion: the persistence of effusion for more than 3 months from the onset, along with the absence of acute infection.

### Diagnostic tests

All countries included in this study recommend the use of otoscopy, pneumatic otoscopy and tympanometry in the diagnosis of OME in children. However, the CPG from the USA recommends the use of 678 or 1000 Hz tympanometry for children aged <7 months and 1000 Hz tympanometry for children aged <6 months. These recommendations reflect that the middle ear of infants has a lower resonance frequency than that of older children.^22^

Additional testing is mostly supported by other CPGs, including pure-tone audiometry, acoustic stapedial reflex testing, otoacoustic emissions and the tuning fork test. The CPGs from several countries including Japan, Korea, France and England outline specific ages and conditions under which other hearing tests may be more suitable than standard pure-tone audiometry. There is a consensus that alternative testing is recommended for children aged <4 years^23^ and patients who cannot cooperate. These alternatives include subjective tests such as conditioned auditory response audiometry, behavioural observation, visual reinforcement and play audiometry as well as objective tests.

The French CPG considers nasal endoscopy an essential part of assessing OME, while the Korean CPG recommends additional tests such as operating microscopy, otoendoscopy and otomicroscopy. Otomicroscopy is reported as the most sensitive and specific approach for diagnosing OME but is difficult to perform and not widely used in primary care settings.^24^ These tests are not mentioned in the Malaysian CPG. The Japanese CPG also recommends temporal bone imaging, as mastoid cell development is related to the onset and prognosis of OME. However, the French CPG considers imaging for OME to be of minimal value. The mastoid cell system develops through pneumatisation in children until the age of 18 years, but this process can be hampered by middle ear disease, as normal middle ear mucosa is a prerequisite for proper pneumatisation. One study showed that the volume and surface of the mastoid cell system in children with OME were significantly smaller than those in healthy children of the same age. However, some studies demonstrated that this application was not specific to OME.^25^

### Management criteria

Watchful waiting is not mentioned in the French and British guidelines. However, the other CPGs recommend that active observation be implemented for 3 months before surgical intervention, along with education to minimise the impact of hearing loss during this period. This approach applies only to patients who are not considered to be at a high risk.

For conservative management, intranasal steroids may be used for OME in cases of concurrent allergic rhinitis and adenoid hypertrophy for a duration of less than 6 weeks. This approach is mentioned in the Malaysian CPG but is not used in Japan, Korea, the USA, Scotland, England and France. In Korea, the long-term effectiveness of intranasal steroids is considered insignificant. Conversely, the only approved oral medication in Japan is carbocysteine.

The reason why most countries have decided to actively observe patients with OME may be that the causative factor is either viral or allergy-related rather than bacterial. This supports the idea that watchful waiting with active observation for 3 months is preferable to the use of antibiotics, as OME often resolves spontaneously. However, if it does not resolve after this period, surgical intervention is recommended.^26^

The Malaysian CPG does not recommend antihistamines for OME, and there are valid reasons for this. It has been noted that antihistamines not only prolong ear effusion but also cause their own set of side effects.^27,28^ Systemic and intranasal steroids have similar efficacy; however, if needed, intranasal steroids are preferred due to their lower bioavailability and fewer side effects.

### Surgical intervention

In Malaysia, myringotomy with ventilation tube insertion is considered only if OME has persisted for more than 3 months with conductive hearing loss of more than 25 dB with or without structural changes to the tympanic membrane and middle ear. In Korea, myringotomy is considered if a patient presents with bilateral conductive hearing loss of more than 40 dB, while the threshold for intervention is over 20 dB in other cases.^6,17^

In England and Japan, myringotomy is recommended for hearing loss of more than 25 dB, but in Scotland, myringotomy is not considered if a patient does not present with other problems such as speech and language difficulties, developmental delays or behavioural problems.^14,15,29^ In the USA, myringotomy is performed if a patient is at risk or has a type B tympanogram in either ear.

Adenoidectomy is considered in Malaysia if a patient presents with hypertrophied adenoids abutting the torus tubarius. In France, due to the risk of bleeding, this surgery is performed only in children below the age of 4 years who exhibit symptomatic adenoid hypertrophy. Conversely, adenoidectomy is not recommended in England and Japan unless a patient presents with symptoms of adenoid hypertrophy and surgery is performed after an initial myringotomy.^5,6,14-16^ In the USA, adenoidectomy is indicated in cases of nasal obstruction and chronic adenoiditis, in addition to OME.

### Management of children with special needs including those with a cleft palate and Down’s syndrome

The management of children with special needs is not specifically mentioned in most CPGs. In Malaysia, myringotomy and ventilation tube insertion are performed in patients with cleft lip and palate at the time of palatal repair. In the USA, screening for OME and hearing loss should continue throughout childhood, including after palatal repair.

According to the Malaysian CPG, myringotomy and ventilation tube insertion are conducted for children with Down’s syndrome after weighing the risks and benefits in the presence of OME alone. In England, these procedures are performed in children with a cleft palate following primary closure after detailed ontological and audiological assessments. They may also serve as an alternative to hearing aids for children with a cleft palate who have OME and persistent conductive hearing loss.^15^ However, there are no recommendations for myringotomy and ventilation tube insertion for children with Down’s syndrome. Hearing assessments are performed for children with Down’s syndrome, cleft lip and cleft palate from birth and then regularly every 6 months in England and Malaysia. Hearing amplification may be considered in cases of mixed or moderate hearing loss in both conditions.^19^ In the Korean CPG, the management of children with special needs is not mentioned, but specialists are suggested to carefully investigate the medical and developmental status of at-risk children.^17^ Conversely, the Malaysian CPG clearly states the surgical management of children with special needs with or without symptoms of OME. In the USA, the CPG recommends ongoing screening for OME and hearing loss throughout childhood, including after palatal repair, and suggests hearing assessments for children with Down’s syndrome every 6 months starting at birth. The other four countries do not include the management of children with special needs in their CPGs.

The discrepancies noted in the CPGs between the selected countries are due to several factors. Variations in healthcare delivery and funding mechanisms lead to different guideline priorities. Patient expectations also influence medical practices and recommendations.^30^ Additionally, economic differences impact the availability of medical technologies and treatments.^31^ Different regulatory requirements and health policies also contribute to these discrepancies.^32^ Varying degrees of involvement from government and medical societies shape guideline development.^33^ Further, variations in disease prevalence and health risks necessitate different clinical approaches, as do differences in medical education and training programmes.^30^

## Conclusion

CPGs for OME management in Japan, Malaysia and Korea share many parallels with CPGs in Scotland, England, France and the USA. However, Asian CPGs are superior to Western CPGs in a majority of areas and levels of evidence.

## Recommendations

- Hearing screening programmes are important for detecting congenital or early hearing loss in children.
- Hearing loss should be detected in newborn babies before 3 months of age, and interventions should be provided no later than 6 months of age.
- Otoscopy, pneumatic otoscopy and tympanometry are recommended for the diagnosis of OME in children.

## Review protocol registration

The study was approved at the 202nd IMU Joint Committee on Research and Ethics Meeting.

Project ID number IS385/semester 6, on 16 July 2020. Bukit Jalil, Kuala Lumpur, Malaysia.
